# Antibiotics promote aggregation within aquatic bacterial communities

**DOI:** 10.3389/fmicb.2014.00297

**Published:** 2014-07-01

**Authors:** Gianluca Corno, Manuela Coci, Marco Giardina, Sonia Plechuk, Floriana Campanile, Stefania Stefani

**Affiliations:** ^1^Microbial Ecology Group, Institute of Ecosystem Study, National Research CouncilVerbania, Italy; ^2^Laboratory of Molecular Microbiology and Antibiotic Resistance, Department of Bio-Medical Sciences, University of CataniaCatania, Italy

**Keywords:** antibiotic resistance, experimental ecology, aquatic bacteria, ecological interactions, aggregation

## Abstract

The release of antibiotics (AB) into the environment poses several threats for human health due to potential development of AB-resistant natural bacteria. Even though the use of low-dose antibiotics has been promoted in health care and farming, significant amounts of AB are observed in aquatic environments. Knowledge on the impact of AB on natural bacterial communities is missing both in terms of spread and evolution of resistance mechanisms, and of modifications of community composition and productivity. New approaches are required to study the response of microbial communities rather than individual resistance genes. In this study a chemostat-based experiment with 4 coexisting bacterial strains has been performed to mimicking the response of a freshwater bacterial community to the presence of antibiotics in low and high doses. Bacterial abundance rapidly decreased by 75% in the presence of AB, independently of their concentration, and remained constant until the end of the experiment. The bacterial community was mainly dominated by *Aeromonas hydrophila* and *Brevundimonas intermedia* while the other two strains, *Micrococcus luteus* and *Rhodococcus* sp. never exceed 10%. Interestingly, the bacterial strains, which were isolated at the end of the experiment, were not AB-resistant, while reassembled communities composed of the 4 strains, isolated from treatments under AB stress, significantly raised their performance (growth rate, abundance) in the presence of AB compared to the communities reassembled with strains isolated from the treatment without AB. By investigating the phenotypic adaptations of the communities subjected to the different treatments, we found that the presence of AB significantly increased co-aggregation by 5–6 fold. These results represent the first observation of co-aggregation as a successful strategy of AB resistance based on phenotype in aquatic bacterial communities, and can represent a fundamental step in the understanding of the effects of AB in aquatic ecosystems.

## Introduction

The occurrence of antibiotics (AB) in aquatic environments is of major concern because of potential spread of AB resistance and of ecosystem alteration (Levy, 1992).

Natural AB are constantly produced by microorganisms and their presence in the environment in very low concentrations was largely underestimate as a potential key factor in controlling ecological interactions (Gullberg et al., 2011). The increased use of AB, and the production of new generations of semi-synthetic AB raise even more concerns about their effect on the natural bacterial communities. Pharmaceuticals, including AB, are only partially eliminated in wastewater treatment plants, therefore residual amounts can reach surface waters or groundwater, where these bioactive compounds potentially impact on natural microbial communities (Hirsch et al., 1999; Czekalski et al., 2012). AB load has been quantified for a number of water bodies and water treatment plants all over the world, demonstrating a strong correlation between human activities (urbanization, farming) and the amount of AB released in the water (McArdell et al., 2003). Although most water treatment plants can reduce by an order of magnitude the concentrations of several AB by sorption transfer to sewage sludge (Giger et al., 2003), AB loads in the range of 5–10 μg L^−1^ have been detected in a number of rivers in different highly anthropized areas around Europe in the last decades (Richardson and Bowron, 1985; Hirsch et al., 1999; Giger et al., 2003). The problem of AB in aquatic environments has been rather underestimated with the consequence of a poor policy on the control of the AB releases (Ternes, 1998; Sarmah et al., 2006) resulting in the discovery of a number new AB resistances developed in aquatic environments (Baquero et al., 2008; Kümmerer, 2009).

Currently used bactericidal or bacteriostatic AB, which are thus released in the environment, are grouped in three main modes of antimicrobial action: ABs acting against the bacterial membrane or cell walls, (e.g., β-lactam), AB targeting protein synthesis through the ribosomal subunits (e.g., tetracycline), and ABs which interfere with the nucleic acid synthesis (e.g., fluoroquinolones) (Sengupta et al., 2013). These groups of AB are nowadays considered within the principal contributors to emergence and maintenance of new resistances within the natural bacterial communities, resulting in direct risk for human health (McArdell et al., 2003). A number of studies found large pools of genes involved in the development of resistances to clinically relevant AB within the complex bacterial communities of aquatic and terrestrial environments (D'Costa et al., 2006; Pruden et al., 2006), where horizontal gene transfer can promote their rapid spread and maintenance. Resistances generally covered the whole sets of AB tested, confirming the rapid adaptability of natural communities, not only to the natural AB but also to new synthetic ones: in 2005 the resistance of over 400 strains library isolated from soils to natural erythromycin (introduced in 1952) was of 27% while the resistance against the semisynthetic telithromycin (FDA approved in 2004) was already of 17% of the strains (D'Costa et al., 2006). This leads to the consideration of environmental bacterial communities exposed to anthropic impact as significant repository of AB resistances.

A number of possible factors can promote the development of a resistance to a specific AB in low dose, and bacterial communities usually exposed to high selection pressure in the environment, developed peculiar features (e.g., broad phylogenetic diversity, phenotypic plasticity, presence of peculiar strains within the rare species, competition pressure favoring rapid evolution) that makes them extremely feasible toward AB resistance spread.

Recently, ideal reservoirs of AB resistances in waters were identified by Drudge and coworkers (Drudge et al., 2012) in water flocs, microparticles composed by a number of tens to thousands of bacterial cells belonging to different species and eventually grown around an organic substrate, i.e., “marine snow” (Alldredge and Silver, 1988) and “freshwater snow” (Grossart and Simon, 1993). This can be attributed to the chemical richness of the flocs themselves coupled to the proximity of bacterial population that lives aggregated around the particle. The flocs can represent a barrier against AB penetration, reducing its concentration toward the center of the aggregate, and at the same time, because of proximity, can promote horizontal gene transfer and thus enhance AB resistance spread within the clustered bacterial community. These particular microenvironments are also well studied in oceanography and theoretical ecology, as they represent ideal hot-spots for bacterial production and organic matter degradation (Azam and Malfatti, 2007; Corno et al., 2013), with enhanced ecological interactions between organisms and complex food-webs in a spatially limited habitat.

It can be guessed that the presence of low doses of AB in waters, promotes not only resistance but, as more immediate response, other kinds of adaptations of the bacterial community, potentially involving alterations of the fitness and of species composition, with unpredictable effects on the stability of the system itself.

In order to deepen our knowledge on the ecological effects of AB in natural water bodies, we performed the first experimental study where different doses of AB triggered a natural bacterial community under controlled laboratory conditions. The bacterial community was designed in order to reproduce a very simplified non-AB resistant freshwater planktonic community. In continuous culture mimicking lake water conditions, bacteria were exposed to a cocktail of three antibiotics chosen from the most commonly used ones in Europe. We measured bacterial productivity and fitness, community composition, and phylogenetic distribution. Furthermore, we tested the degree of adaptation to AB of single strains and communities (i.e. acquisition of AB resistance) in order to assess the ecological impact of AB in concentrations comparable with anthropized waters in Central Europe on the bacterial community.

## Materials and methods

### Selection of isolates and antimicrobial susceptibility tests

Four freshwater bacterial strains isolated from European lakes have been used for this study: *Aeromonas hydrophila* strain GC035, *Brevundimonas intermedia* strain GC044 and *Micrococcus luteus* strain GC037 have been isolated from Lake Zurich (Switzerland), while *Rhodococcus* sp. strain NO0007 has been isolated from Lake Maggiore (Italy). Partial 16SrDNA sequences of each strain are deposited in Genbank (accession nrs. KJ409640-43). The community composed by the mixture of these strains reproduces an extremely simplified bacterial community of a classical deep European oligo-mesotrophic lake: *A. hydrophila* (Gammaproteobacteria) and *B. intermedia* (Alphaproteobacteria) are common in freshwater and in particular conditions can dominate lakes communities (Farmer et al., 1992; Zwart et al., 2002), while *M. luteus* and *Rhodococcus* sp. (Actinobacteria) despite rather frequently found in freshwaters are always limited to very little numbers and non-significant relative abundances (Newton et al., 2011).

The four strains were preliminary subjected to antimicrobial susceptibility test to the AB further used in this study, namely levofloxacin, tetracycline, and imipenem (Oxoid, Milan, Italy) by disc diffusion method, according to the Clinical and Laboratory Standards Institute CLSI (2008). Because published breakpoints for the species used are missing, the interpretive standards of the diameter zone were applied as follow: non-Enterobacteriaceae or nonfastidious Gram-negative rods were used to interpret the diameter zone values for *B.intermedia* and *A. hydrophila; Staphylococcus* sp. for *M. luteus*, and *Corynebacterium* for *Rhodococcus* sp. (CLSI, 2008 and EUCAST 2014). The same procedures and interpretative standards were used for the determination of antimicrobial susceptibility of the four strains isolated after the experiment in continuous culture.

### Continuous culture design

Bacterial strains were pre-cultivated for 3 days in Artificial Lake Water medium (ALW, Zotina et al., 2003). Aliquots from each pre-culture were then used to prepare the experimental community, which was inoculated into the chemostat.

**Figure 1:**
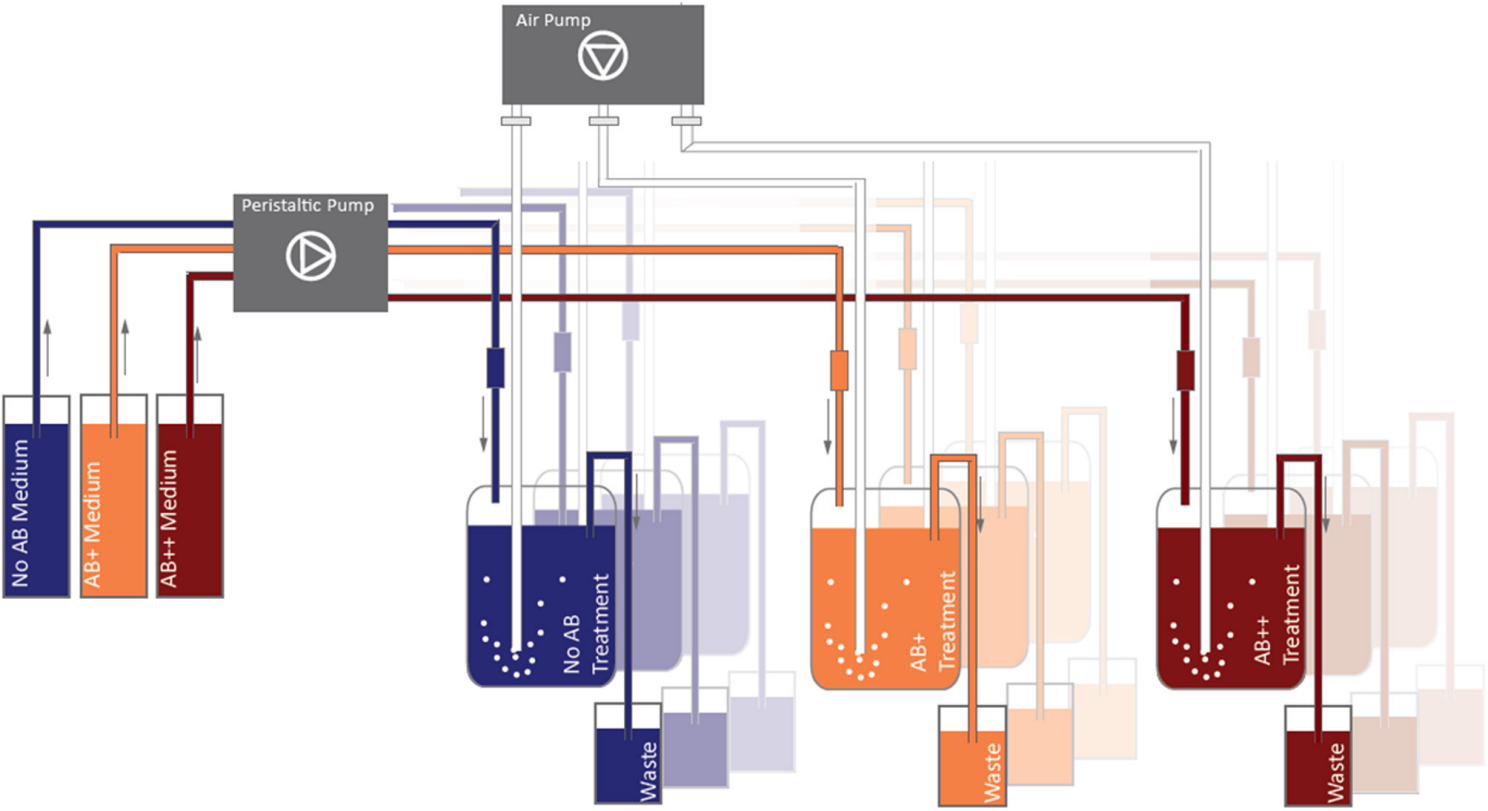
**Continuous culture design**. Substrate was continuously renewed (0.2 d-1) through peristaltic pumps, aeration (fine bubbling) was performed in order to ensure a full mixing of the water in the vessel and to prevent the deposition of detritus.

The main experiment was carried out in a one-stage continuous cultures system consisting of 9 reactors (Figure 1); three parallel chemostat vessels were run for each treatment (NO AB, AB+, AB++). The chemostat was assembled in a climate chamber (18 ± 1°C) with a night-day period of 12 h and ran for 25 days. The 9 vessels were filled with 750 ml of ALW medium enriched with 10 mg glucose L^−1^ as the sole C-source. New medium was continuously pumped in the vessels by a multichannel peristaltic pump (Watson-Marlow 205S) from three 20 L reservoirs, in order to achieve a dilution rate of *D* = 0.2 d^−1^. The vessels were aerated from the bottom by fine bubbling with sterile air (Figure 1).

AB impact on the bacteria was tested at different concentrations of the same AB cocktail, composed by an equal mixture of tetracycline (Sigma-Aldrich, CAS number 60548), imipenem (Sigma-Adlrich, CAS 74431-23-5; β-lactam, subgroup carbapenems), and levofloxacin (Sigma-Adlrich, CAS 100986854; fluoroquinolon) to a final concentration of 12.5 μ g AB L^−1^ (treatment AB+), and of 125 μ g AB L^−1^ (treatment AB++). Treatment NO AB, without antibiotics, was used as control. The AB mixtures were added directly to the AB+ and AB++ 20 L reservoirs. Concentration AB+ mimes the natural concentration of AB in polluted effluxes by waste water treatment plants in anthropized areas in Central Europe (Kümmerer, 2009), while concentration AB++ represent a concentration 10 times higher than the highest reported in nature. The selection of the three ABs used for the cocktail is consistent with the major AB groups nowadays consumed in Western countries (Food and Drug Administration report 2011—FDA Annual Report on Antimicrobials Sold or Distributed for Food-Producing Animals in 2011; UCM338170).

About 18 ml of the assembled freshwater community was inoculated into each of the 9 reactors to obtain a final concentration of 1.0 × 10^6^ cells ml^−1^ (composed by 0.25 × 10^6^ cells ml^−1^ of each strain). After an acclimation of 48 h without dilution, the system was switched on and the experiment started. Daily samples (5 ml/vessel) were collected for the analysis of bacterial abundance; weekly samples (25 ml/vessel) were taken for the analyses of community composition and phenotypical distribution.

### Bacterial abundance and phenotypical diversity

Bacterial cell numbers were determined from 0.5 ml formalin-fixed (2% final concentration) samples stained with 4′,6-diamidino-2-phenylindole (DAPI) (Porter and Feig, 1980), filtered onto 0.2 μm polycarbonate filters, and counted by epifluorescence microscopy (Axioplan, Zeiss). At least 400 bacterial cells were counted per sample. Different phenotypes were classified on the base of the bacterial social behaviors: free living single cells, microcolonies composed by few aggregated cells (generally belonging to the same strain), and larger aggregates composed by several clustered individuals belonging to different species (Corno et al., 2013). Images for the sizing of aggregates and microcolonies were captured with a DP72 high resolution camera (Olympus) and evaluated by image analysis (ImagePro Plus, Media Cybernetics).

Aggregates size was approximated by determining the maximal Feret dimension (Fe_max_) of single cells cluster as detected on DAPI stained filters. Aggregates were grouped into size classes each 10 μm. Cell clusters composed by at least 5 cells, with Fe_max_ <10 μm were considered as microcolonies. The relative importance of the aggregated cells within the bacterial community was obtain by the estimation of their average number for aggregate for each size class, then mediated to cover the aggregates size distribution for each sample (Corno et al., 2013). In detail, the average cells number per aggregate was estimated by the median values for the corresponding aggregate size class and the average bacterial cell sizes, as obtained from epifluorescence microscopy. The reliability of the estimation was tested by comparing the organic carbon content of the bacterial community (estimated per bacterial cell per strain according to Loferer-Krössbacher et al. (1998) and the direct measurement of POC in the same sample. Briefly, the relative proportion of each strain in the sample, the specific amount of C per cell of each strain, and the difference between the total measured POC and the POC in single free living cells, were used to estimate the amount of C in the aggregates, then related to the different size classes (Corno et al., 2013). In order to reduce the potential approximation error in the different size classes we merged our results in the general group “Aggregates.”

### Bacterial community composition

The composition of the bacterial community was analyzed by catalyzed reporter deposition-fluorescence *in situ* hybridization (CARD-FISH) coupled with fluorescence microscopy. Weekly collected samples were fixed with freshly prepared buffered PFA (1% final concentration) and then concentrated on 0.2 μm polycarbonate filters (GTTP, Millipore). Filters were rinsed twice with sterile phosphate buffered saline (PBS), air-dried and stored at −20°C until further processed. The following probes were used to determine the relative proportions of specific bacterial populations: ALF968 for Alphaproteobacteria (Glöckner et al., 1998), GAM42a (mixed with the corresponding competitor probe) for Gammaproteobacteria (Manz et al., 1992), and HGC69a for Actinobacteria (Roller et al., 1994). CARD-FISH was performed according to Sekar et al. (2003). The relative proportion of each strain was then counted by epifluorescence microscopy. Actinobacteria were additionally subdivided within the two strains used in this study, by visual recognition: while *Rhodococcus* sp. cells are rods of different length, *M. luteus* cell shape is always coccoid with constant dimensions, thus they are morphological easily distinguishable by epifluorescence microscope.

### Post continuous-culture re-isolation and analysis

At the end of the experiment, each bacterial strain was isolated from each treatment on ALW agar plates in order to be tested for potential acquired AB resistance. *Rhodococcus* sp. strain could not be isolated from treatments AB+ and AB++; the clonal strain of the inoculum was used instead. Purity of the isolated strains was checked by CARD-FISH: only cultures with 100% of positive hybridization rate were then used in further experiments. Single cultures from the different isolates were tested for antimicrobial susceptibility as described above. Single cultures and re-assembled communities (composition is described below) were also tested for growth in 96 well plates: 200 μl culture in ALW medium enriched with 20 mg L^−1^ of Glc, inoculum concentration 1 x 10^6^ bact ml^−1^ in triplicate for each experimental treatment. For example, *A. hydrophila* isolated from NO AB treatment was inoculated in triplicate in NO AB, AB+, and AB++ wells. The disposition of the treatments and the distribution of the strains in the plates was randomized. Bacterial communities were re-assembled by mixing the strains in identical proportion to reproduce the same initial conditions as in the chemostats. Plates were incubated (same conditions than for the chemostat) for 48 h, radially shaken for 30 s every 30 min and growth was measured every 24 h as optical density (OD) of each well with a plate reader Glomax Multi-detection System (Promega) cleaned of the blank signal and other potential noises.

### Statistical analyses

In order to evaluate the significance of the difference in the median values between groups the Wilcoxon-Mann-Whitney test was applied. To test for statistical differences with the expected median value within a single time series a *t*-test was performed, after testing for normal distribution of the series. All analyses were performed by using software JMP10 (SAS Institute Inc.).

## Results

### Antimicrobial susceptibility tests in pre and post treated isolates

The resistance antibiotypes of the four isolates—before and after the continuous culture experiments—are shown in Table 1. All strains resulted to be susceptible to the tested antibiotics with the exception of *B. intermedia* which showed intermediate resistance for levofloxacin. The correlation of the resistance antibiotypes allowed us to exclude potential pre-existing competitive advantages for single strains once exposed to AB.

**Table 1:** **Results of antimicrobial susceptibility tests by disk diffusion method**.

**Bacterial strains**	**Zone diameter values in mm (results)**		
	**IMP**	**TET**	**LEV**
*Aeromonas hydrophila* GC035	34 (S)	28 (S)	34 (S)
*A. hydrophila* (NO AB)	33 (S)	29 (S)	33 (S)
*A. hydrophila* (AB+)	34 (S)	29 (S)	34 (S)
*A. hydrophila* (AB++)	34 (S)	29 (S)	34 (S)
*Brevundimonas intermedia* GC044	47 (S)	38 (S)	18 (I)
*B. intermedia* (NO AB)	47 (S)	38 (S)	18 (I)
*B. intermedia* (AB+)	47 (S)	38 (S)	18 (I)
*B. intermedia* (AB++)	47 (S)	38 (S)	18 (I)
*Micrococcus luteus* GC037	33 (S)	23 (S)	22 (S)
*M. luteus* (NO AB)	33 (S)	23 (S)	23 (S)
*M. luteus* (AB+)	33 (S)	23 (S)	23 (S)
*M. luteus* (AB++)	33 (S)	23 (S)	23 (S)
*Rhodococcus* sp. NO007	55 (S)^*^	40 (S)	26 (S)
*Rhodococcus* (NO)	55 (S)^*^	40 (S)	26 (S)

Zone diameter values of the four isolates before the experiments /first row for each strain) and of those re-isolated from the 3 different treatments (NO AB, AB+, and AB++). The results of the interpretation are indicated in brackets (S, susceptible, R, Resistant, I, Intermediate;

* *values ≥ 55 are off scale)*.

*For disk zone diameter interpretation CLSI (2008) and EUCAST (2014) standards were used, considering the values for non-Enterobacteriaceae or nonfastidious P.aeruginosa, S.aureus and Corynebacterium sp. for A. hydrophila and B. intermedia, M. luteus and Rhodococcus sp., respectively*.

### Bacterial abundance and community composition

**Figure 2:**
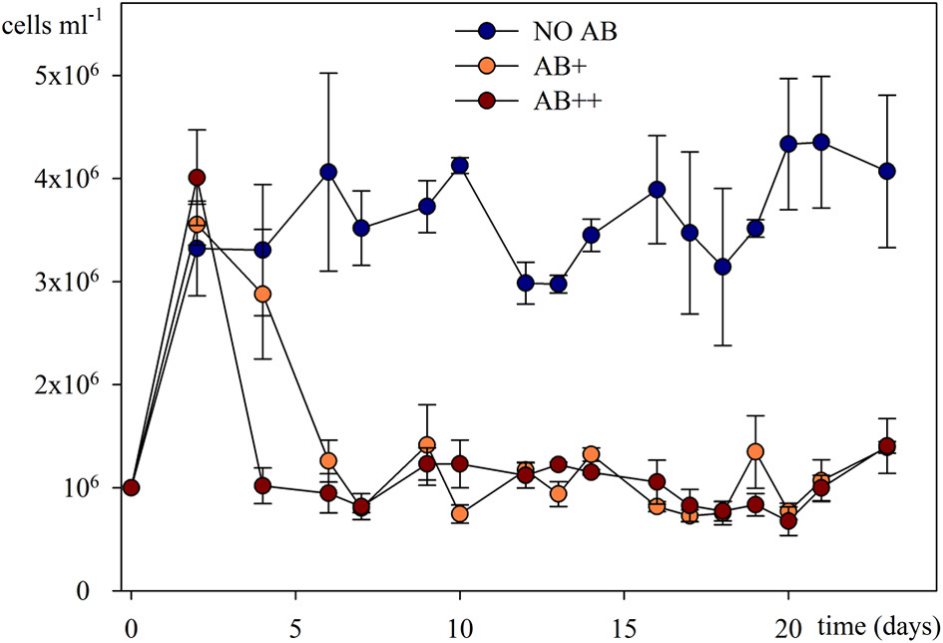
**Bacterial abundances in continuous culture vs. time**. Overall bacterial numbers are given as mean of three replicates (± *SD*) for each of the three treatments.

A steady community of about 4 × 10^6^ bact ml^−1^ characterized the treatment NO AB already from day 2, without significant fluctuations through the whole experiment (*P* = 0.532), while in treatments AB+ and AB++ the overall community abundance rapidly reduced by 75% (to about 1 × 10^6^ bact ml^−1^) independently by the AB concentration, and remaining then constant to the end of the experiment (Figure 2). No significant differences in numbers were detected between the treatments AB+ and AB++ considering the period 4–24 days (*P* = 0.836).

**Figure 3:**
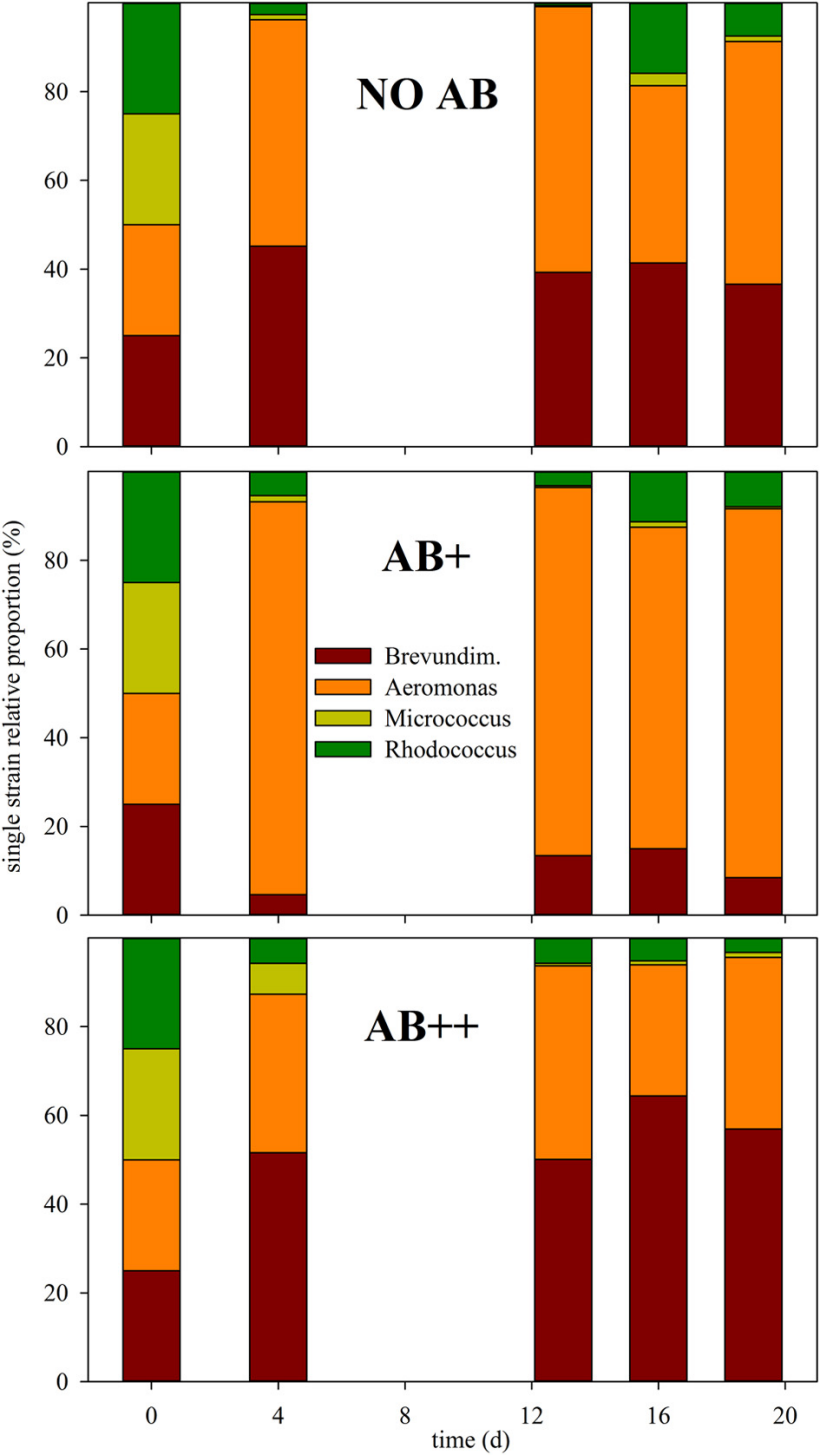
**Relative proportion of each strain in continuous culture vs. time**. The relative proportion of each strain is given as mean of three replicates at day (d) 0, 4, 13, 16, and 20, for each of the three treatments (from top to bottom): NO AB, AB+, AB++.

Community composition did not have important variations during the experiment after day 2 (Figure 3), either. The four strains could be detected in every sample on each date, thus extinction events were not detected for any AB concentration. Communities resulted either dominated by *A. hydrophila* (AB+), *B. intermedia* (AB++), or by an almost equal proportion of the two strains (NO AB), that together always achieved a relative abundance over 90% of the total bacterial number. The two Actinobacteria rapidly reduced from 50 to less than 10%, with a relative predominance of *Rhodococcus* sp. in all the treatments.

### Phenotypical distribution

**Figure 4:**
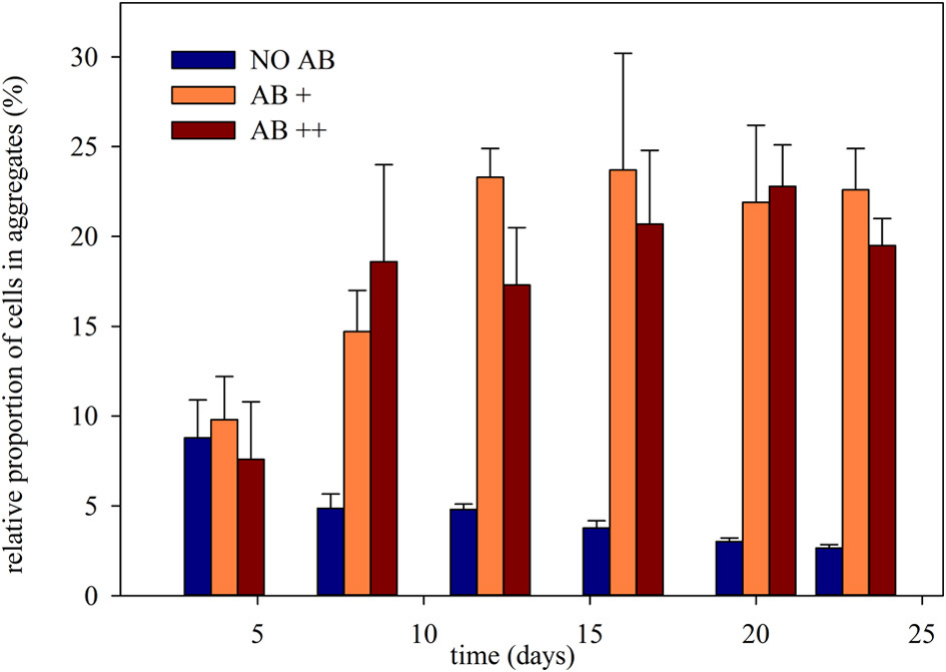
**Relative proportion of cells clustered in continuous culture vs. time**. The relative proportion of aggregated cells is given as mean of three replicates (± *SD*) for each of the three treatments.

The weekly assessment of the relative proportion of free living single cells, microcolonies composed by a few cells (in average up to 25), and larger cell aggregates (Figure S1) showed different aggregational behavior of the bacterial community once exposed to AB (Figure 4). Clustered cells (microcolonies and aggregates) at the beginning of the experiment never exceed 10% of the overall community. In treatment NO AB their proportion constantly decreased to less than 3%, while in treatments AB+ and AB++ they rapidly rose to 20-25%, keeping then constant up to the end of the experiment. Again, there was a significant effect of the AB (*P* < 0.001 for both treatment comparison: NO AB vs. AB+ and vs. AB++), but no significant differences between the two treatments with AB (*P* = 0.394).

### Re-growth of adapted strains

Bacterial strains isolated from the different vessels at the end of the continuous culture experiment were then tested for the development of AB resistance, potentially acquired while exposed to AB in the chemostat (Table 2). Zero or negative values between time 24 and 48 in the newly isolated single culture demonstrated that none of the three strains tested (*B. intermedia, A. hydrophila*, and *M. luteus*) could grow when re-inoculated in AB enriched media, independently by the AB concentration and by their treatment of origin. At this stage it resulted impossible to recover *Rhodococcus* sp. from the continuous culture treatments with AB, where *Rhodococcus* sp. was present only within co-aggregates. *Rhodococcus* sp. already demonstrated a very slow growth on agar, then, it is likely that in plates it suffered the competition by most growth-effective strains, and thus got outcompeted. For this reason and for consistency, in the post-continuous cultures experiments we used the same *Rhodococcus* sp. strain we used at the beginning of the experiment for the inoculum in the chemostat.

**Table 2:** **Growth of re-isolated strains and reassembled communities**.

**Growth T24–T48 (Absolute OD values)**			
**Bacterial strains/communities**	**NO AB**	**AB+**	**AB++**
*Brevundimonas* (NO AB)	**0.083**	0.023	0.009
*Aeromonas* (NO AB)	**0.068**	−0.011	0.005
*Micrococcus* (NO AB)	**0.040**	−0.019	−0.003
*Rhodococcus* (NO AB)	0.020	−0.007	0.008
*Brevundimonas* (AB+)	**0.049**	0.036	0.002
*Aeromonas* (AB+)	**0.054**	−0.009	0.004
*Micrococcus* (AB+)	**0.047**	−0.011	−0.002
*Brevundimonas* (AB++)	**0.056**	0.011	0.016
*Aeromonas* (AB++)	**0.050**	0.013	0.005
*Micrococcus* (AB++)	0.022	−0.003	0.002
*Community* (NO AB)	**0.112**	−0.001	0.002
*Community* (AB+)	**0.103**	**0.032**	**0.042**
*Community* (AB++)	**0.104**	**0.056**	**0.046**

*Differences in optical density (OD) between growth time 24 and 48 h of bacterial cultures isolated from the different treatments at the end of the continuous cultures and of the relative mixed reassembled communities. In brackets it is mentioned the origin of the strain/community, while bold numbers indicate statistically significant growth (P < 0.01, student t-test)*.

These results are supported by the susceptibility confirmed with disk diffusion methods after the continuous culture experiment (Table 1). At the same time all strains responded positively to the growth in NO AB medium, without significant differences between the same strains isolated from different treatments.

**Figure 5:**
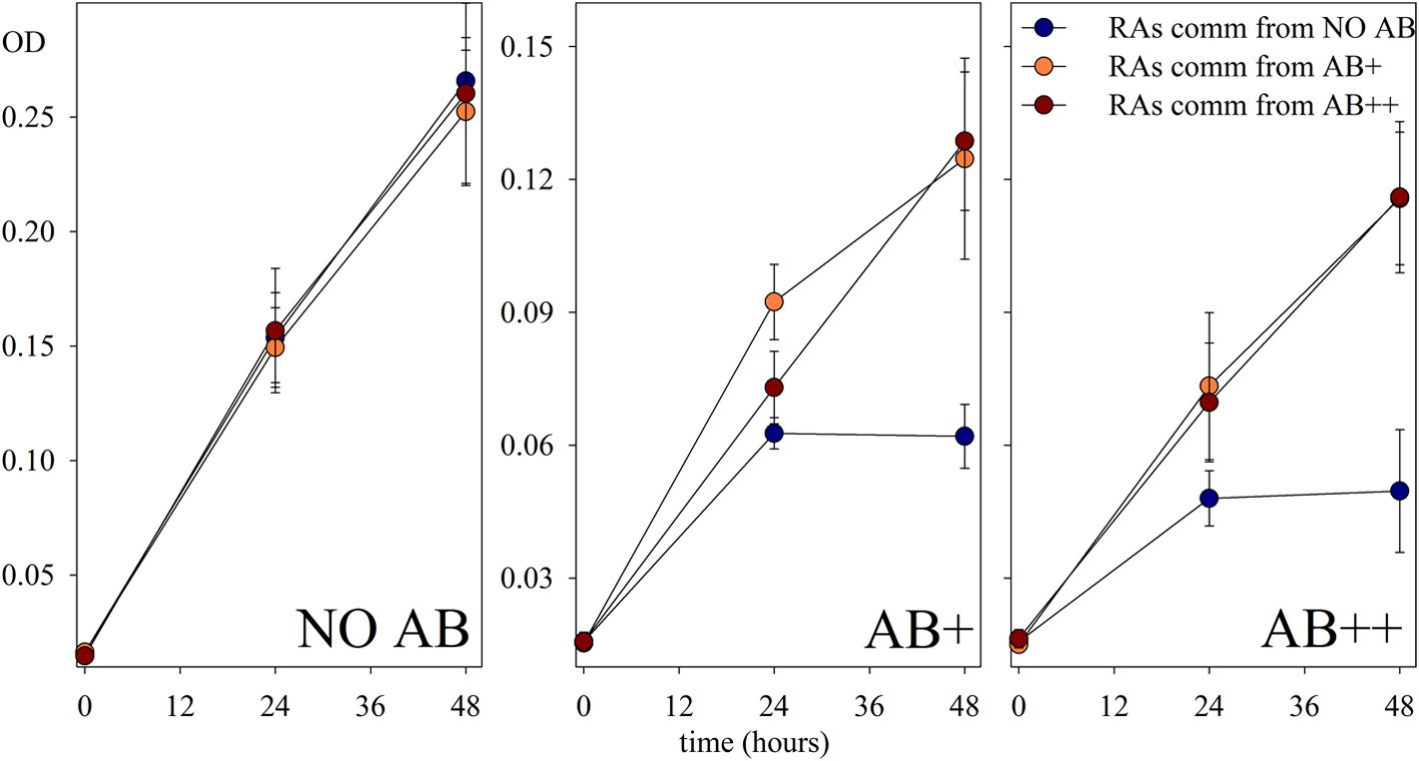
**Growth of the reassembled communities**. Growth of communities reassembled (RAs comm) from the experimental strains at the end of the continuous culture treatments, and preferentially re-exposed to each experimental condition (NO AB, AB+, AB++). Values are in optical density (OD) of each sample, and are given as mean of three replicates (± *SD*) for each of the treatments.

The re-assembled communities, composed by an equal mixture of the strains isolated at the end of the continuous culture experiment, performed as expected in treatments NO AB, again without significant differences between communities isolated from different treatments with or without AB. In contrast, a significant growth of the communities composed by strains exposed to AB during the continuous culture experiment was detected when those were grown in presence of AB (Figure 5). The acquired adaptation observed in these communities is confirmed by the concomitant failure of communities from the NO AB treatment that, once exposed to AB, did not grow as the single strains that composed them. Also in this respect, the impact of AB caused a comparable response independently by the AB concentration in the medium.

## Discussion

The assessment of the overall sub-lethal (or the sub-inhibitory) concentration of antibiotics for a natural bacterial community is basically impossible, as too many bacterial species and even subspecies can have different sensitivity to different AB. Moreover, the sensitivity of the different species composing the community can change in time and space with changing ecological parameters, community composition, and evolutionary history. At the same time, recent studies demonstrated that the response to AB in low concentrations by bacterial communities in anthropized open waters, or in waste water treatments, can significantly differ from the response of those in clinical environments (Gagneux et al., 2006).

The design of a simplified bacterial community, tested under experimentally controlled conditions, reduces the complexity of the natural environment and allows speculations at the community level, excluding a number of ecological interactions (i.e., viral lysis, predation) that would exponentially increase the number of variables to be taken into account (Horňák and Corno, 2012), reducing our possibility to correctly evaluate the actual effect of the AB in the system. The four strains we selected for this study rather well represent a model community of a large Central European lake, with strains common in every water body and sometimes very abundant (*A. hydrophila* and *B. intermedia*) and others less successful in waters but anyway almost always represented within the “rare biosphere” (*sensu* Pedros-Alio, 2007).

The impact of AB was immediately clear in terms of productivity of the system (Figure 2) but, interestingly, the concentration of AB was not causing significant differences in this respect, whereas it resulted in a different selection in terms of bacterial community composition (BCC; Figure 3). In detail, the fitness of the bacterial community, measured in number of new cells produced (thus, for a chemostat, in cells abundance) was highly affected by the presence of AB, and the reduction in bacterial number (about 75% in treatments AB+ and AB++) was independent from the AB concentration. This result is not in accordance with the general theory, stating that being in low concentrations, the impact of AB in natural environment is negligible (Waksman, 1961). In this respect, a number of studies (reviewed by Sengupta et al., 2013) demonstrated a clear impact of low doses of AB in nature while our study demonstrated that AB can have an impact on the productivity of natural bacterial communities not only when in low doses, but that this impact can be comparable to higher dosages, raising our concerns for the release of AB into nature.

Remarkably, the reduction of fitness had only a limited correspondence with alterations of the overall ecological success of the single strains (Figure 3). The two strains, *A. hydrophila* and *B. intermedia*, which dominated the community without AB, still kept having a clear supremacy also in presence of AB. Moreover the “rare” Actinobacteria did not gain any advantage by the reduction of potential competition for resources. *A. hydrophila* appeared to be the most successful strain in communities NO AB and AB+, while at higher AB dosage *B. intermedia* resulted to be the most abundant genotype. This can be related to a intermediate susceptibility of *B. intermedia* to levofloxacin in high dosages, or to a significant increment of the inhibitory activity of AB toward *A. hydrophila*. In any case, the modifications occurred in the BCC demonstrated that, despite the buffering capabilities of mixed bacterial communities can limit the impact of external factors, the presence of AB, even in low concentration, can modify the structure of the bacterial community. Reduced resistance and potential reduced resilience of the bacterial community can promote the success of allochtonous bacterial strains (e.g., pathogens, enhanced when AB are the disturbing factor) which would otherwise be eliminated by the means of competition.

Although modifications in the genotypic composition of the community were only partially affected by the presence of AB, the phenotypic distribution drastically changed. When exposed to AB the bacterial community switched from a free living single cells dominated community (treatment NO AB) to communities where almost 1 bacterial cell out of 3 clustered in aggregates with other cells (Figure 4). Since the experiment was conducted in a continuous culture system, with a constant efflux, this increment in proportion within the community has to be related to an effective clustering activity, thus in a significant competitive advantage for clustered cells in comparison to the free living ones. Large co-aggregates composed by up to 500 cells belonging to 2–4 different strains, as well as smaller microcolonies composed by only one strain rapidly appeared in presence of AB and, again, their proportion was comparable in treatments exposed to different AB concentrations. It can be speculated that the resistance strategy developed in our system by typical freshwater strains is based on the formation of aggregates, that ensure a AB free environment for the cells embedded into them, and that this strategy is thus equally efficient at AB+ and AB++ antibiotic concentrations. Co-aggregates and microcolonies represent a well-studied phenotype (or a specific “social behavior”) in planktonic bacteria: aggregation can protect bacteria from predation (Corno and Jürgens, 2008), from starvation (Hahn et al., 2000), and can reduce competition and raise the productivity (Blom et al., 2010), thus preserves diversity and reduces the extinction risk in some peculiar cases (Horňák and Corno, 2012).

Comparable to biofilms on surfaces (Costerton et al., 1999), bacteria forming aggregates are surrounded by different forms of self-synthetized hydrated exopolymeric matrix effectively reducing the diffusion of the AB because of to the reduced permeability of the aggregate itself. Co-aggregation is thus a fast-developing winning strategy for planktonic bacteria once exposed to AB in low to intermediate concentrations, by forming particular niches where, possibly, the effect of AB is reduced by dilution, and the competition for resources can be less limiting (Corno et al., 2013). Similar observations have been recently published by Haaber et al. (2012) on pure cultures of *S. aureus*: under AB stress the formation of large clusters was accompanied by an increment in productivity. The authors found direct evidences of an increase in tolerance toward stressors like AB for *S. aureus* once aggregated. A very similar conclusion can be reached by our study on a more complex bacterial community, where treatments exposed to AB selected for bacteria with enhanced tendency toward aggregation, thus less sensitive to antimicrobials. It would be reasonable to consider the aggregational state, already present in reduced proportion at the beginning of the experiment mainly as microcolonies, not as an acquired feature, but simply a most competitive state in presence of AB. Nevertheless, our results on the growth of recombined communities (Figure 5) show that communities reassembled with strains from treatments AB+ and AB++ perform significantly better than the one from NO AB treatment, once re-exposed to AB. The first reassembled communities somehow kept a “memory,” possibly because they were isolated from cells belonging to aggregates. This observation implies that in nature, where clustering formation is common for many aquatic bacterial strains, aggregation can be a “ready to use” phenotypical adaptation for bacterial communities once exposed to AB in low concentrations, and that these microenvironments should deserve a deeper and more focused attention in the research of AB resistances in aquatic ecosystems. In the 96-wells plate we could test the short-term response in presence of AB of re-isolated strains and re-assembled communities from AB treatments: the difference in the growth curves of the re-assembled communities from treatments with AB in comparison with those of the “non-adapted” ones were already highly significant after 48 h from the beginning of the incubation. While the latter dropped in abundance, the “adapted” communities were still in exponential growth. We do not consider the enhanced OD values recorded for the “adapted” communities as an evidence for potential resistance but as a confirmation that the adaptation observed was due to coexistence of the different strains, and that it is an acquired feature. We can speculate that the observed differences in OD are given by aggregate formation, as we did not measured the phenotypical composition of the communities within the wells. Our opinion is that after the initial spin of the bacterial growth due to the fresh amount of organic C available, the AB had its impact on the less adapted communities, and the difference become evident after 48 h. A similar trend in growth has been detected in chemostat, too. In comparison to the analyses of communities in chemostat, with OD in wells there is a loss in the power of the analysis, excluding the possibility for a discrimination between active and inactive cells: the missed growth of many cultures after 48 h could be accounted to a stable community, but also to a stable number of inactive cells, thus to a dead community.

Finally, the composition of almost the totality of the aggregates we observed in this study was multispecific. Not only, single strains isolated from treatments under AB pressure, did not perform better than the control strains once exposed to AB, while the same strains in the reassembled communities did. We can thus suggest that only through multispecific co-aggregation the community gains an advantage against the antimicrobial action. The complexity of the interactions within the clustered cells raised enormously, resulting in the formation of a specific microenvironment where, through proximity and species interactions, the resistance of the single bacterial cells against AB rose.

We can thus suggest co-aggregates as ideal environments for fast adaptations to AB presence in aquatic systems. Our results, following the observations of Costerton et al. (1999) of biofilms as ideal environments for the development of AB resistances and pathogen preservation in clinical environments, and of Drudge et al. (2012) on the enhanced ability for aquatic bacteria to share AB resistance genes in flocs compared open waters, highlight the importance of aggregational states in the understanding of the ecology of aquatic ecosystems exposed to AB.

AB contamination of global water supplies is raising, increasing the risk of the development of clinically important AB resistance in these environments in the close future, with this study we offer one of the first attempts to get deeper into the ecology of bacterial communities exposed antimicrobials in low doses.

### Conflict of interest statement

The authors declare that the research was conducted in the absence of any commercial or financial relationships that could be construed as a potential conflict of interest.
