# Supplementary material for: Antibiotics promote aggregation within aquatic bacterial communities
Source: Front Microbiol. 2014 Jul 1;5:297. doi: 10.3389/fmicb.2014.00297 (PMC4077313; doi:10.3389/fmicb.2014.00297)
Supplement: Supplementary file 1 [file Presentation1.ZIP › S1 legend.docx]

**Figure S1: Microphotographs of different bacterial phenotypes in continuous cultures.** A: Large aggregate from treatment AB+ in DAPI, all cells in the aggregate are fluorescently labelled, B: The same aggregate as in A, but labelled with CARD-FISH probe for γ-proteobacteria, only *A. hydrophila* is visible, and dominates the aggregate, C: Rare aggregate composed mainly by Actinobacteria from the treatment AB++, D: Very large aggregate from treatment AB++, E: Bacterial community from treatment NO AB, composed almost exclusively by free living bacteria. All images are taken from continuous culture samples at day 16, the white dotted bar in each figure il 20 μm long.
