# Supplementary figures and images for: Antibiotics promote aggregation within aquatic bacterial communities
Source: Front Microbiol. 2014 Jul 1;5:297. doi: 10.3389/fmicb.2014.00297 (PMC4077313; doi:10.3389/fmicb.2014.00297)

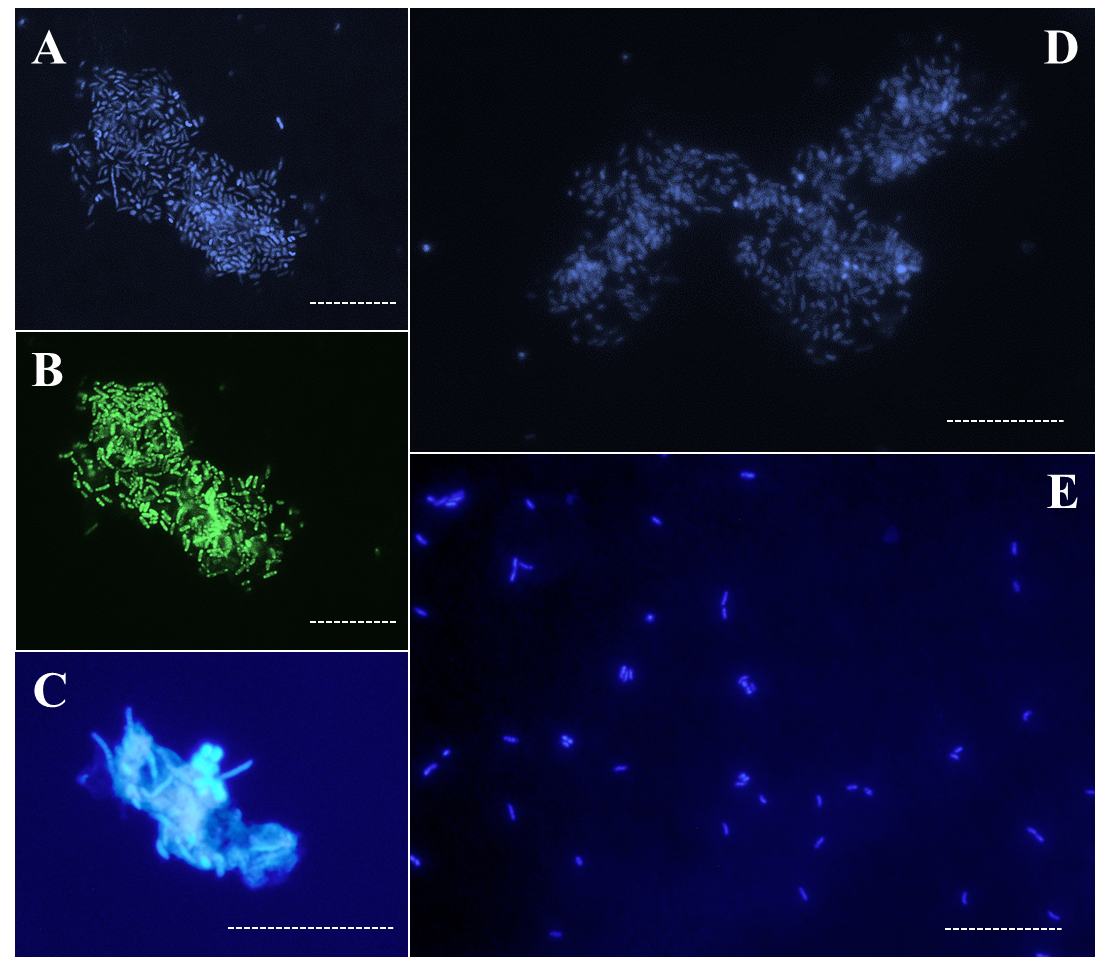

Supplement: Supplementary file 1 [file Presentation1.ZIP › Supp Fig 1.TIF]
